# Projecting the SARS-CoV-2 transition from pandemicity to endemicity: Epidemiological and immunological considerations

**DOI:** 10.1371/journal.ppat.1010591

**Published:** 2022-06-30

**Authors:** Lily E. Cohen, David J. Spiro, Cecile Viboud

**Affiliations:** 1 Icahn School of Medicine at Mount Sinai, New York, New York, United States of America; 2 Division of International Epidemiology and Population Studies, Fogarty International Center, National Institutes of Health, Bethesda, Maryland, United States of America; University of Pittsburgh, UNITED STATES

## Abstract

In this review, we discuss the epidemiological dynamics of different viral infections to project how the transition from a pandemic to endemic Severe Acute Respiratory Syndrome Coronavirus 2 (SARS-CoV-2) might take shape. Drawing from theories of disease invasion and transmission dynamics, waning immunity in the face of viral evolution and antigenic drift, and empirical data from influenza, dengue, and seasonal coronaviruses, we discuss the putative periodicity, severity, and age dynamics of SARS-CoV-2 as it becomes endemic. We review recent studies on SARS-CoV-2 epidemiology, immunology, and evolution that are particularly useful in projecting the transition to endemicity and highlight gaps that warrant further research.

## Introduction

The trajectory of the ongoing Coronavirus Disease 2019 (COVID-19) pandemic remains uncertain; current barriers to containment include asymptomatic transmission [1,2], waning immunity [3,4], emergence of new variants, and, in many countries, both persistent levels of infection and public resistance to interventions such as social distancing and vaccination. Although billions of vaccine doses have already been administered throughout much of the world [5,6], the rapid spread of the Severe Acute Respiratory Syndrome Coronavirus 2 (SARS-CoV-2) Omicron lineage has shown that neither vaccines nor prior natural infection is sufficient to protect against large waves of infections by new variants.

Past experience suggests that after jumping into humans, a zoonotic pathogen may experience different epidemiological trajectories depending on a variety of factors, including its ability to transmit between humans and the effectiveness of control measures [7]. We distinguish between 4 possible paths of an emerging pathogen, including “sporadic spread,” “local or regional spread,” “pandemicity,” and “endemicity.” Sporadic spread is characterized by short chains of human-to-human transmission that quickly die out due to low transmissibility (also called stuttering chains of transmission [8]). This is a feature of pathogens that do not transmit well between humans and have an R0 less than 1, where R0 is the basic reproduction number that measures the number of secondary cases generated by an infected individual in a completely susceptible population. Classic examples of sporadic spread include influenza A/H5N1, monkeypox, and Middle East respiratory syndrome (MERS) (outside of the hospital settings where transmission is amplified) [8]. For emergent pathogens with a higher human-to-human transmission potential (R0>1), a period of local or regional spread can be observed followed by (temporary) disappearance in human populations due to rapid intervention, as illustrated by Ebola or SARS-CoV-1. An important feature of these pathogens is that, while R0>1, control is possible by contact tracing and other nonpharmaceutical interventions because the period when the pathogen transmits from infected to susceptible individuals coincides with the symptomatic phase of the disease [7]. Unfortunately, around 50% of SARS-CoV-2 transmission occurs before symptom onset, or from fully asymptomatic individuals, making it particularly difficult to stop transmission without strict interventions such as social distancing [9]. An alternative outcome for an emergent pathogen above the critical threshold of R0>1 is pandemicity, which is characterized by rapid, global spread in the face of little or no preexisting population immunity. This is best exemplified by the recent history of HIV, pandemic influenza, and SARS-CoV-2. After a global pandemic, a transition to endemicity is generally expected. Here, we define endemicity as the long-term (multiyear) persistence of a pathogen in a population at a steady annual level of infection.

Transition to endemicity has been observed several times for pandemic influenza in the last hundred years, while many of the vaccine-preventable childhood infections, such as measles and mumps, likely shared a similar past centuries or millennia ago [10]. In the endemic period, and in the absence of new interventions, we expect regular periodicity in the occurrence of outbreaks, predictable seasonal patterns, and a mean rate of infection that fluctuates minimally over time. We contend that endemicity is the most plausible route for SARS-CoV-2 in the foreseeable future, with SARS-CoV-2 poised to eventually become the fifth endemic seasonal coronavirus (HCoV) along with HKU1, NL63, OC43, and 229E [11,12]. In this article, we attempt to project some of the epidemiological, evolutionary, and immunological characteristics of SARS-CoV-2 outbreaks in the endemic phase.

### Epidemiological theory of pathogen invasion

Mathematical models have long been used to predict the trajectory of epidemics, particularly in periods of dynamical changes such as the emergence of a new pathogen (e.g., pandemic influenza or a new dengue serotype), the rollout of new vaccines (e.g., rotavirus) [13], or changing demography (e.g., declining birth rate leading to fewer susceptible individuals) [14]. Invasion of new pathogens into naive populations that have no immunological memory to the pathogen, and their persistence in those populations, follows a predictable pattern [15–17]. Initially, the pathogen’s arrival causes several large waves of infections that burn through a fraction of susceptible hosts of all ages, depending on control interventions [11] and R0. It is unlikely that SARS-CoV-2 will burn itself out after the initial waves have ended: Most countries have persistent levels of infection, and despite vaccination, the risk of reintroduction and onward spread is high in a post-pandemic world. Resurgences have occurred even in countries that have had relatively successful public health responses to the pandemic such as China, Australia, and New Zealand, in part due to international travelers sparking outbreaks in areas with high population susceptibility and the emergence of immune escape variants like Omicron [18].

Mathematical models predict that future waves of infections will be more moderate in size and sweep through remaining susceptibles, including individuals who have escaped infection and vaccination, and new births [12]. In addition, loss of immunity to infection due to natural decay of protection over time and the emergence of immune escape variants will replenish the pool of susceptibles. The rate of influx of new susceptibles will determine the periodicity of outbreaks in the endemic state, with slower replenishment leading to less frequent outbreaks. Further, environmental forcing and changing seasonal contact patterns (e.g., school calendars) is expected to modulate transmission and determine outbreak seasonality. In the long-run, SARS-CoV-2 outbreaks will likely be entrained into annual winter cycles as is the case for the 4 endemic human coronaviruses (HCoVs), 229E, OC43, HKU1, and NL63 [12,15], and many other respiratory pathogens [19]. Multiyear epidemic cycles (e.g., every other year or every 3 years) are seen across a range of strongly immunizing infections, such as measles and pertussis, as well as several infections that confer partial immunity such as rotavirus and respiratory syncytial virus (RSV) [19–22]. Yet, these pathogens are characterized by notably slower evolutionary rates than SARS-CoV-2. As a result, influenza and seasonal coronavirus outbreaks, characterized by marked annual periodicity, are more likely illustrations of what future SARS-CoV-2 outbreaks may look like.

### Past influenza pandemics

In projecting the epidemiological switch from pandemicity to endemicity for SARS-CoV-2, it can be useful to look back at past pandemics. Historical influenza pandemics provide a template for the rapid emergence and global spread of new viruses, followed by continued periods of circulation lasting from 10 to 50 years. We will focus here on 2 relevant events: the 1918 and 2009 pandemics. The 1918 A/H1N1 pandemic is particularly interesting because of its severity and scale; the global impacts of the first 2 years of the 1918 pandemic are comparable to the first 2 years of COVID-19 in some areas [23]. However, a full understanding of the epidemiology of the 1918 pandemic remains elusive as we do not know which influenza strain was circulating before 1918, obscuring any patterns of preexisting population immunity. In contrast, the 2009 A/H1N1 influenza pandemic had a moderate health impact when compared to the 1918 pandemic or COVID-19; yet, a wealth of epidemiological, immunological, and evolutionary data documents the transition to endemicity.

**Fig 1:**
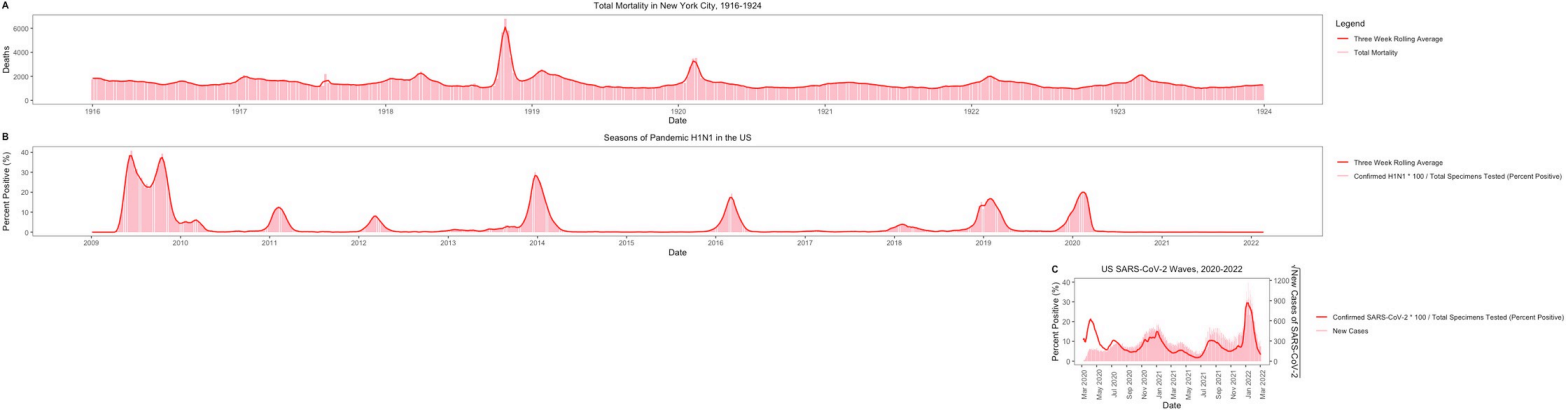
Waves of influenza A/H1N1 and COVID-19 during initial invasion and subsequent circulation. Data sources: (A) Olson and colleagues [24]; (B) Centers for Disease Control and Prevention, National Center for Immunization and Respiratory Diseases (NCIRD) [25]; (C) Ritchie and colleagues [6]. Coronavirus Disease 2019, COVID-19; Severe Acute Respiratory Syndrome Coronavirus 2, SARS-CoV-2.

Both the 1918 and 2009 influenza A/H1N1 pandemics were marked by the occurrence of several closely spaced waves in the first year of circulation, reminiscent of the 2020 COVID-19 patterns (see Fig 1 for a comparison of all 3 pandemics) [24,25]. In the first year of SARS-CoV-2 circulation, successive pandemic waves have resulted from changes in contact patterns due to social distancing, masking, and winter aggregation. In the second year of the pandemic, after population immunity had risen through vaccination and infection, subsequent waves were primarily fueled by the emergence of more transmissible variants. For pandemic influenza, the major elements that appeared to cause successive waves include seasonal environmental factors, school closures and reopenings [26,27], and in the case of the 1918 pandemic, restrictions on mass gatherings [28].

All influenza pandemics since 1918 were marked by a shift of infections and deaths towards younger ages, compared to seasonal influenza [29,30]. For the 2009 pandemic, high infection rates were reported for children and adults up to ages approximately 50 years old, likely due to a combination of protective effect of prior immunity among seniors, who had seen similar influenza viruses in their past [29], and increased contact rates among susceptibles of younger ages. Although schools were temporarily closed in the early days of the 2009 A/H1N1 pandemic in a few countries (e.g., Mexico, Hong Kong, Peru) [31], these interventions were quickly lifted once it became clear that the A/H1N1 virus caused limited mortality. Importantly, schools remained open for the duration of the main pandemic wave in fall 2009 in the Northern Hemisphere.

The age shift in infections, cases, and deaths that accompanies the switch from pandemic to endemic influenza operates over several years. Data from Denmark suggest that it took about 7 years to return to the typical age profile of seasonal influenza mortality after the emergence of the 1918 pandemic virus [32]. In recent years, age patterns in influenza cases have been driven by differences in immune imprinting to A/H1N1 viruses and cocirculating A/H3N2 viruses, with recent epidemics retaining a propensity to infect younger age groups compared to pre-2009 pandemic levels [33].

### Interpreting age-based analyses

A key determinant of the age profile of future epidemics, and hence their potential health burden, is the age structure of the susceptible population and the force of infection. The force of infection is defined as the risk of becoming infected for a typical susceptible per unit of time, providing a measure of the level of circulation of a pathogen in the community. In settings with higher forces of infection, we would expect a younger mean age at first infection, because the risk of getting infected in each year of life after birth is high. This is a well-described epidemiological phenomenon seen in infections with lifelong or persistent immunity, such as measles, dengue, or poliovirus [34], which have classically provided a foundational model for the expected age distribution of an emerging virus.

While the switch from initial invasion wave to endemicity happened a very long time ago for measles and polio, 2 diseases that are now on their way to control or eradication, a more recent transition has been observed for particular dengue serotypes in Latin America. Thus, dengue is a useful model for understanding the drivers of age dynamics, and the connection between force of infection and endemicity. Decades of dengue surveillance and seroprevalence studies have illustrated that as dengue circulates over time and becomes endemic in a population, the force of infection decreases and the age at which individuals in a population acquire dengue-specific immunity rises [35–37]. However, time in circulation and preexisting immunity are not the only contributing factors; demographic change, such as declining birth rates, and outbreak control can also decrease transmission intensity and in turn affect age distributions [35–38]. In a pediatric cohort study in Managua, Nicaragua, for example, the age at which children were first infected more than doubled between 2004 and 2015, due to the introduction of a new dengue serotype in the 1990s, combined with a decrease in birth rate and increase in life expectancy that together lowered transmission intensity [36]. In contrast, a 40-year study in Bangkok, Thailand, where dengue has been established for much longer, revealed only a 1-year increase in mean infection age [37].

Based on epidemiological and immunological data collected since the beginning of the COVID-19 pandemic, it is clear that the age distribution of SARS-CoV-2 infection does not follow the classic age profile of other pathogens. Young children appear to have immunological or biological protection against infection compared to other age groups, which was particularly evident prior to widespread vaccination of teenagers and adults. The reasons why children may have lower susceptibility to SARS-CoV-2 have yet to be elucidated. Possible cross-protection from the endemic human coronaviruses, which frequently infect children, has been explored and refuted [39,40]. Some studies have also suggested that the expression of key viral entry-associated receptors, such as ACE2 and TMPRSS2, increases with age, and that children may express fewer receptors and/or receptors with lower affinity for SARS-CoV-2 in the respiratory epithelium [41–43]. Other analyses, however, have disputed these findings [44,45]. In addition to lower susceptibility than adults, children may also enjoy reduced disease severity upon infection. The airway epithelia of children may express a higher baseline level of interferon-response genes than adults, which may reduce viral spread [46]. Additionally, children have a greater diversity of T-cell receptors, potentially enabling them to recognize and eliminate unknown pathogens to a greater extent than adults whose immune response is more focused on previously encountered infective agents [46]. Additional biological differences may affect the age patterns of infection; for example, waning of immunity against infection may be more pronounced in older age classes, as is observed for seasonal coronaviruses and RSV [47,48].

Vaccination, waning immunity, and evolution are important driving forces of changes in age dynamics. Infections are expected to shift towards younger age groups as more adults are vaccinated—a pattern that has materialized in the first year of the SARS-CoV-2 vaccination program [49]. In contrast, antigenic drift, the gradual genetic evolution of immunologically important viral antigens due to random mutation, would increase susceptibility in all age groups and shift infections towards a broader range of ages, as seen for influenza [33]. With a higher rate of antigenic drift, and in turn greater immune escape, more individuals with prior immunity can experience reinfection, compared to a scenario of low antigenic drift. With a high rate of antigenic drift, reinfections can occur even in age groups who have experienced multiple infections and tend to be older. Conversely, a slower rate of antigenic drift (less immune escape) would preferentially affect age groups whose prior exposure to the virus is less frequent, concentrating infections in younger age groups. We see a signature of this phenomenon in the age distribution of different influenza subtypes, where influenza A/H3N2 has a faster rate of antigenic evolution than influenza B, with A/H3N2 cases reported to be older on average than influenza B cases [50]. Taken together, HCoVs and SARS-CoV-2 observations [48,51] support that immunity to SARS-CoV-2 infection will wane rapidly (although there are differences between immunity acquired from natural infection and vaccination), while protection against severe disease will be maintained over longer periods [51].

Unfortunately, we cannot fully rely on the age distribution of well-studied pathogens such as measles, dengue, and influenza to predict a future shift in the epidemiology of SARS-CoV-2. Viral evolution, waning immunity, and the introduction of vaccines in younger age groups will each play a role in determining the relationship between age and susceptibility to infection and disease. In the early months of the COVID-19 pandemic, when vaccines were unavailable, children generally appeared to be less susceptible to infection and to present with milder cases. While it seemed originally that children and adolescents infrequently served as index cases [52,53], increased public health surveillance has shown that transmission from these age groups can and does occur [54,55]. In the United States, we saw more children hospitalized with the Delta variant than with previous strains, but this was most likely due to the sheer amount of Delta circulating and children’s lack of vaccine-based protection rather than from an increase in susceptibility or severity in children [56]. We saw similar increases in hospitalization rates in children following the emergence of the Omicron variant [57]. While disease in children is generally less severe than adults, a viral mutation that increases the susceptibility of children to infection and/or severe illness could pose a serious threat both to children and to the population at large. Additionally, the estimate of 80% prevalence of long-term symptoms (fatigue, headache, attention disorder, and shortness of breath) [58] in SARS-CoV-2-infected individuals might also encourage parents and guardians to vaccinate their children if given the opportunity.

### Age distribution of seasonal coronaviruses

**Fig 2:**
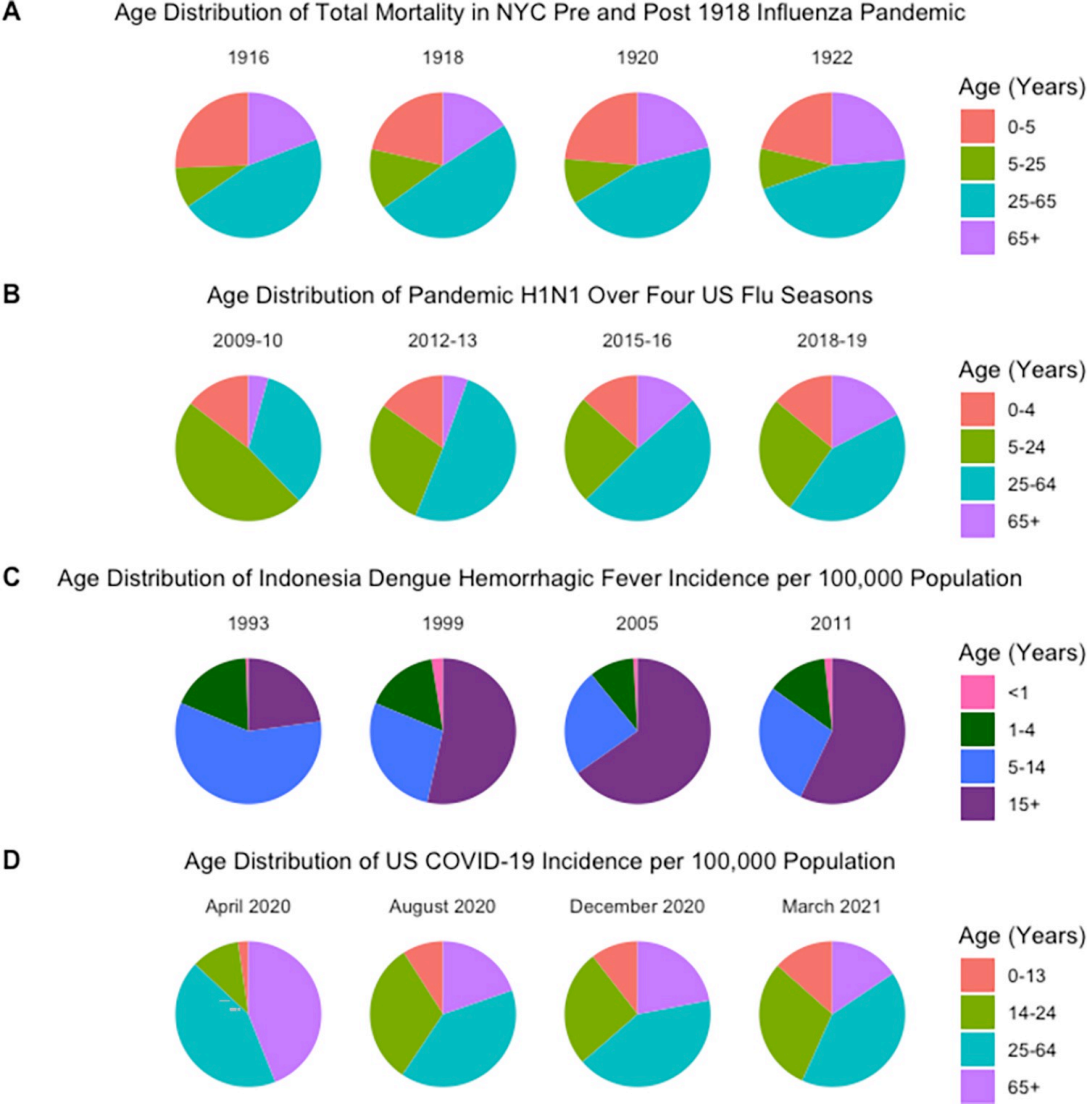
Changing age distributions of influenza A/H1N1, dengue, and COVID-19 during the transition from pandemic to endemic. Data sources: (A) Olson and colleagues [24]; (B) Centers for Disease Control and Prevention, National Center for Immunization and Respiratory Diseases (NCIRD) [25]; (C) Karyanti and colleagues [38]; (D) Centers for Disease Control and Prevention [128]. Coronavirus Disease 2019, COVID-19.

**Fig 3:**
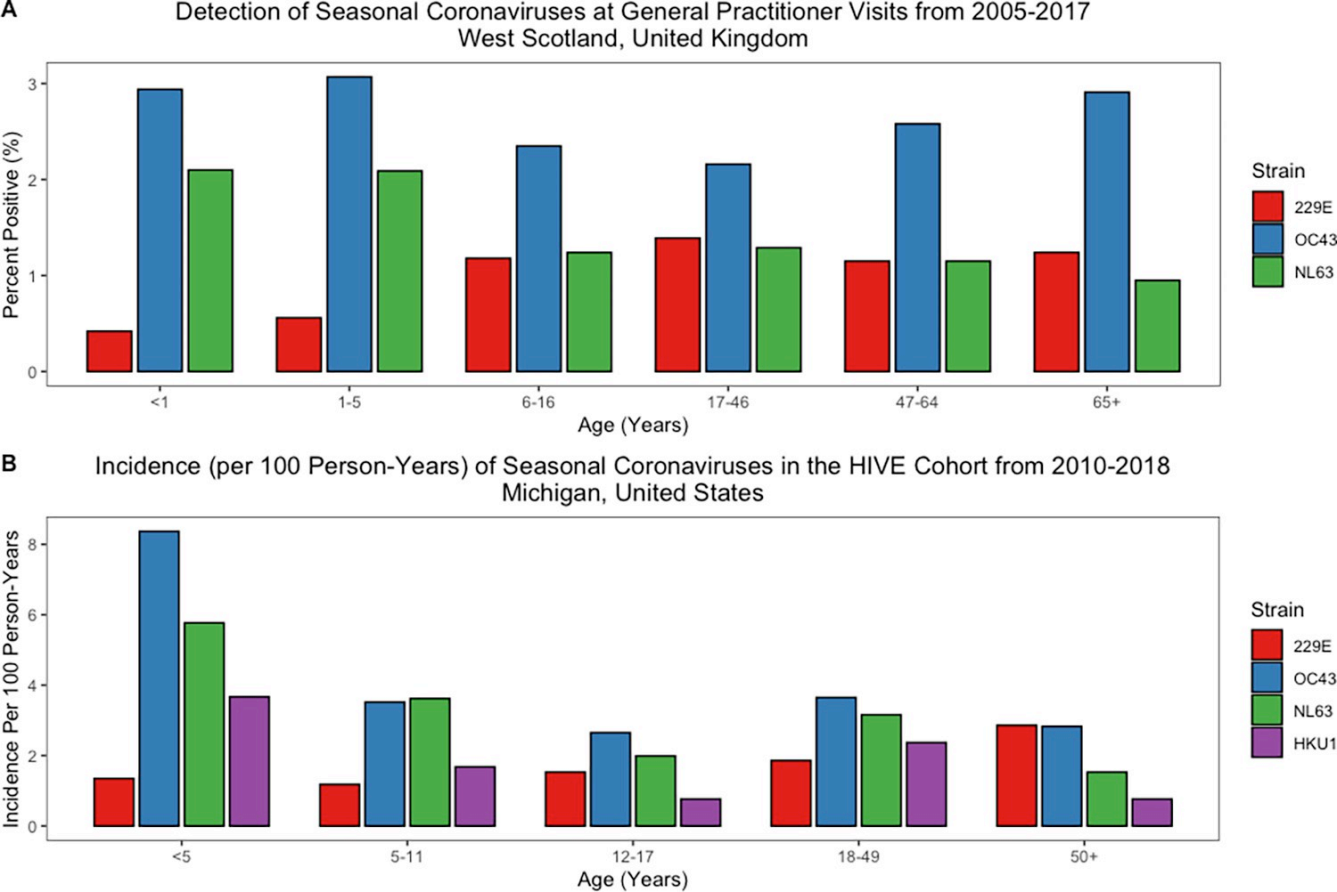
Detection of seasonal coronaviruses in 2 cohort studies, broken down by strain and age group. Data sources: (A) Nickbakhsh and colleagues [61]; (B) Monto and colleagues [60]. HIVE, Household Influenza Vaccine Evaluation.

The age distribution of endemic SARS-CoV-2 infections cannot be fully predicted, although the 4 seasonal coronaviruses that have been established in humans for decades or more provide useful examples (Fig 2). In contrast to SARS-CoV-2, the seasonal coronaviruses (HCoV strains 229E, OC43, NL63, and HKU1) discovered in the 1960s and early 2000s, are thought to predominantly infect young children and generally cause mild respiratory disease [59,60]. Yet, the relationship between age and infectivity in the seasonal coronaviruses is still not entirely transparent; 2 studies published in 2020—one following a cohort of households in Michigan and one conducting diagnostic surveillance in the West of Scotland—show that incidence of seasonal coronavirus infection does necessarily decline with age (Fig 3). The Household Influenza Vaccine Evaluation (HIVE) study, which followed a cohort of between 890 and 1,441 individuals annually from 2010 to 2018, detected a total of 993 seasonal coronavirus infections; HCoV-229E appeared most frequently in adults over 50 years old, while the other strains most frequently infected children under 5 [60]. After age 5, however, incidence was relatively constant across age groups. The West Scotland surveillance study, which tested respiratory samples among 12,628 patients presenting to their general practitioner, showed relatively similar age patterns to the HIVE cohort for strains HCoV-229E and HCoV-NL63; here, however, incidence of HCoV-OC43 was similar across age groups, including infants and children under 5 [61]. Both studies show that there are likely more infections in adults than previously thought; however, neither study includes data on asymptomatic cases, which may occur more frequently in children and affect transmission rates. A single cross-sectional serology study exists, suggesting a more classical age-dependent pattern of infection gradually increasing up to age 20 years [62]. It would be beneficial to design larger prospective HCoV cohorts in the future, particularly from birth, to address questions about duration of immunity and reinfection.

The epidemiology and age distribution of the invasion waves of seasonal coronaviruses—i.e., when they first spilled over to humans—is unknown, but would be particularly useful to project the epidemiological consequences of the switch from pandemicity to endemicity in SARS-CoV-2. The establishment of seasonal coronaviruses would make for an excellent subject of archeo-epidemiology, a branch of epidemiology centered on understanding the epidemiology of ancestral pathogens, using previously unearthed data sources such as church records and genealogy records [63]. Notably, in the wake of the COVID-19 pandemic, the hypothesis that the 1889 Russian influenza pandemic may have been an HCoV-OC43 pandemic has gained traction [64]. Further, phylodynamic analyses of seasonal coronavirus sequences could also contribute to date the putative emergence of different HCoV serotypes. If we can advance our understanding of how the seasonal coronaviruses, particularly HCoV-HKU1 and HCoV-OC43 (2 betacoronaviruses, the same genus as SARS-CoV-2) and HCoV-NL63 (a coronavirus using the ACE2 receptor like SARS CoV2), adapted to exist long term in human populations, we may be able to better predict and prepare for the effects of an endemic SARS-CoV-2.

### Immune response to SARS-CoV-2

#### Immune response after primary infection or vaccination

A critical determinant of the endemic patterns of SARS-CoV-2 infection is the duration, strength, and breadth of immune protection. As the SARS-CoV-2 virus has only been circulating in the human population since late 2019, we are just beginning to understand how immunity may wane in the face of time and viral evolution. Early concerns that the immune response could be short lived have been moderated by data showing a robust immune response to both infection and vaccination. SARS-CoV-2 infection leads to the production of neutralizing antibodies in the vast majority of individuals, with over 90% of mild to moderate cases mounting high titers of neutralizing antibodies to the spike protein [65,66]. Six [67] and eight [68] months after symptom onset, subjects had only a mild decline in circulating neutralizing titers and a strong memory B cell response to the spike protein that increased during the months following infection before plateauing. Eleven months after mild SARS-CoV-2 infection, convalescent subjects showed evidence of SARS-CoV-2 S protein-specific long-lived bone marrow plasma cells, indicating the development of stable immunity against COVID-19 [69]. Furthermore, strong CD4+ and CD8+ T-cell responses against multiple proteins have been identified in the majority of COVID-19 patients [66,67,70]. SARS-CoV-2-specific follicular helper T cells, critical in establishing memory B cells, were also found in a significant fraction of COVID-19 patients [69]. However, these responses can be heterogeneous; in particular, T-cell immunity decreases with both age (>65 years old) and severe disease [71].

These immunological findings are corroborated by epidemiological studies, indicating that reinfections are rare over a 7- to 8-month period (80% to 85% protection), and when reinfections occur, they are significantly less severe than primary infections [72–74]. It is worth noting, however, that the likelihood of reinfection is increased in older individuals, so that more frequent revaccination may be warranted than for younger individuals. Further, the emergence of Omicron has increased the risk of reinfection across the entire population compared to earlier variants both for previously infected [75] and vaccinated individuals [76], suggesting that antigenic drift will require periodic vaccine updates. While in vitro studies indicate substantial decrease in neutralization titers against new variants, vaccine protection remains around 88% against symptomatic disease with the Delta variant, and 45% with Omicron, in the immediate weeks after a second dose [77]. Vaccine boosters increase effectiveness against symptomatic infection to 90% for Delta and 60% for Omicron; yet, protection against SARS-CoV-2 symptoms wanes rather rapidly after primary vaccine course and boosters [78]. Importantly, all studies point to a higher and longer-lasting level of protection against hospitalization and death, including for Omicron. In particular, vaccine breakthrough cases have milder courses of illness relative to cases in immune-naive individuals [78,79].

Data on the duration of immunity to SARS-CoV-2 is available on the timescale of months but is lacking on the scale of years. In the absence of long-term data specific to SARS-CoV-2, we can turn to related viruses to gauge multiyear immunity patterns, including SARS-CoV-1 and the endemic HCoVs. In studies of patients who had survived SARS-CoV-1 disease, T-cell responses were maintained 6 to 11 years after infection, while neutralizing antibodies and memory B cells were undetectable after 6 years [80,81]. Since the SARS-CoV-1 virus only circulated for a short while in humans, it is impossible to know whether the long-term T-cell immune response is protective, or whether other immune mediators would be boosted by periodic reexposure. A different, more temporary, form of immunity is found in one of the seasonal coronaviruses, HCoV-229E, for which challenge studies have generated particularly useful data. Patients who were experimentally infected with HCoV-229E developed short-lived antibodies against the virus, which were undetectable a year later [51]. When these patients were rechallenged with the same strain of virus, the majority of subjects got infected and shed virus, but they did not show symptoms, suggesting an anamnestic immune response. In a recent observational cohort study, reinfections with all 4 seasonal HCoVs were frequent, occurring with a periodicity of 0.6 to 3 years depending on the serotype [82]. Further, preseason antibody titers were not predictive of the risk of reinfection, confirming that sterilizing immunity is very short lived or lacking for HCoVs.

#### Immunological interactions between and within serotype(s)

Although seasonal HCoVs and SARS-CoV-2 are genetically distant, the potential for positive and negative immunological interactions between these viruses has been the subject of some debate, particularly via the phenomena of original antigenic sin and antigenic seniority, 2 terms originally coined for influenza.

Interactions operate when the humoral memory response against 1 set of antigens (e.g., the seasonal HCoV antigens) modulate the immune response to infection by a similar but not identical set of antigens (e.g., SARS-CoV-2 antigens) [83]. This type of immunomodulation may be protective against infection with viruses seen earlier in life—a concept referred to as antigenic seniority, or “back-boosting,” where responses to past antigens are recalled upon reinfection with a different antigen. Original antigenic sin is a version of this concept that singles out the first exposure in life to a pathogen (or set of pathogens, e.g., all coronaviruses). These mechanisms may explain why we see largely protective responses in individuals vaccinated with an mRNA vaccine after a natural SARS-CoV-2 infection [84], or the amplification of a nonprotective immune response. Several studies have observed immunomodulation caused by HCoV antibodies on SARS-CoV-2: As an example, a strong back-boosting effect was reported to conserved but not variable regions of the HCoV-OC43 and HCoV-HKU1 betacoronaviruses spike protein in hospitalized COVID-19 patients [85]. However, it remains largely unclear in which ways circulating HCoV antibodies affect the SARS-CoV-2 immune response [85–87]. Several other studies have indicated that B cell [88] and T-cell responses mounted against a lifetime of exposures to HCoVs can cross-react with SARS-CoV-2 [70,89], yet there is no known cross-protective effect for B cells [85,90] and the protective role of the T-cell response remains unclear.

Immunologic interactions, such as antigenic sin and back-boosting, could also manifest due to sequential exposure with different variants of the same virus, and we are just beginning to observe this phenomenon with SARS-CoV-2. There is evidence that antibody responses mounted to different SARS-CoV-2 variants target different epitopes of the receptor-binding domain [91], but the impact of antigenic modulation on future responses remains unclear. Evidence of back-boosting exists in the context of Omicron over Delta, but remains limited [92]. It is also unknown whether immunomodulation will operate similarly in individuals repeatedly infected by different SARS-CoV-2 variants and in individuals infected post-vaccination (i.e., breakthrough cases). Longitudinal serology, as used in a 2014 study analyzing the long-term “antibody landscapes” of a cohort repeatedly infected with and vaccinated against influenza, would help elucidate the relevance of original antigenic sin, and potentially back-boosting, to SARS-CoV-2 endemicity patterns [93].

An extreme aspect of immune modulation is antibody-dependent enhancement (ADE), most notable in dengue. Dengue has 4 antigenically distinct serotypes (DENV-1-4); while primary infections are generally mild, secondary dengue infections can cause more severe immunological responses potentially leading to shock and high-grade fever [94–96]. Interestingly, while prior infection with any dengue serotype will enhance disease when reinfected by DENV-2, high dengue antibody titers from a prior infection will protect against disease from DENV-1 and DENV-3 [97]. Notably, cross-reactivity between dengue virus and Zika virus, another flavivirus transmitted by *Aedes aegypti* and *Aedes albopictus*, adds more complexity to dengue’s immunologic and clinical picture. While a prior Zika infection appears to enhance disease caused by DENV-2, a prior dengue infection appears to protect against Zika symptoms [97]. Although ADE was originally a cause of concern with SARS-CoV-2 [98], and ADE has recently been observed in vitro in a SARS-CoV-2 model [99], it has not yet been shown in vivo and the relevance to endemicity patterns remains unclear.

While current research cannot fully address the duration of immunity to SARS-CoV-2, or anticipate complex immunological interactions within and between coronaviruses, existing data are hopeful for a long-term protective response against severe disease. However, the protective effects of the immune response against asymptomatic and pauci-symptomatic infections, and viral shedding, as SARS-CoV-2 viruses continue to evolve, remain debated. The amount of viral shedding resulting from reinfections is important as it will determine the contribution of reinfections to transmission, and in turn affect the force of infection and the periodicity of epidemics.

### Viral evolution and antigenic drift

In future years of SARS-CoV-2 circulation, the emergence of new variants via viral evolution, along with waning immunity to infection and new births, will fuel outbreak recurrence. The pace and mechanism of viral evolution in the endemic phase of SARS-CoV-2 will affect the frequency of updates of future vaccines and the age profile of future SARS-CoV-2 epidemics. To anticipate the long-term evolutionary dynamics of SARS-CoV-2, researchers have turned to pandemic influenza, which is seen as a model for the periodic establishment of a zoonotic pathogen in human populations, followed by rapid antigenic drift during the endemic period. The introduction of novel influenza viruses from avian and porcine reservoirs can cause large shifts in genetic and antigenic material, and occasionally cause pandemics in a variety of mammals, including humans. In parallel, endemic human seasonal influenza viruses undergo mutation in their hemagglutinin and neuraminidase proteins, the targets of neutralizing antibodies elicited by natural infection and vaccination, leading to antigenic drift and decreased vaccine efficacy. The process of antigenic drift is particularly relevant for SARS-CoV-2, as the spike protein has high mutational tolerability.

The pace of influenza antigenic drift has been well described. Influenza vaccine contains at least 3 antigens representing the A/H1N1, A/H3N2, and influenza B strains projected to circulate in the coming year. While at least 1 component of the vaccine is updated every 1 to 2 years to account for antigenic evolution, the 2017 to 2018 influenza season marked the first post-pandemic update of the influenza vaccine A/H1N1 component, 8 years after the emergence of the 2009 A/H1N1 pandemic virus [100]. It has been proposed that the evolution of the 2009 pandemic A/H1N1 virus took place in 2 phases after it entered human populations: host adaptation of the virus immediately after its arrival (2009 to 2010) followed by immunological selective pressure beginning in 2014 and leading to a decrease in vaccine efficacy [101].

A similar phenomenon of initial adaptation to a new host followed by antigenic evolution is taking place with SARS-CoV-2, although the process is accelerated relative to influenza. At the beginning of the pandemic, it was assumed that SARS-CoV-2 would evolve more slowly than influenza strains because its mutation rate is approximately 3 times less than influenza [102] due to the presence of an unusual RNA viral proofreading mechanism in coronaviruses [103]. However, SARS-CoV-2 has demonstrated periods of rapid mutation, especially in the S1 domain of the spike protein, with the Delta variant of concern evolving at a rate 5 times that of the HA1 region of A/H3N2 influenza [104].

SARS-CoV-2 has undergone 3 distinct phases of evolution during the pandemic. The first period, from viral emergence to winter 2020, was characterized by the establishment of the D614G spike gene mutation in the majority of circulating viruses and by relative evolutionary dormancy as characterized by lack of change in viral transmissibility, pathogenicity, and antigenicity [105]. The second phase, from December 2020 to November 2021, was marked by the global dissemination of the variants of concern (VOCs) Alpha, Beta, Gamma, and Delta first detected in the United Kingdom (September 2020), South Africa (May 2020), Brazil (November 2020), and India (October 2020), respectively [106]. The mutations associated with these VOCs were concentrated in the receptor binding and N-terminal domains of S1 and have been associated with increased binding affinity for ACE2 and decreased monoclonal antibody neutralization in vitro [105]. Epidemiologically, the second phase was characterized by VOCs with increased transmissibility and viral fitness, but insufficient immune escape to require a vaccine update or to promote reinfection [75]. The third phase was the emergence of the Omicron VOCs (BA.1, BA.2), which had significantly more mutations than previous circulating strains (30 mutations in the spike gene as compared to 8 to 12 mutations for Alpha, Beta, and Gamma), and formed a new antigenic cluster, potentially requiring a vaccine update [107]. The Omicron VOCs are also characterized by greater transmissibility than previous variants, perhaps due to increased tropism for the upper respiratory tract as compared to lung epithelia [108], and significant immune escape, resulting in lower vaccine efficacy [77]. Explanations for the sudden appearance of the highly divergent Omicron clade include evolution in a geographic area where genomic surveillance was insufficient to detect intermediate variants, zoonosis from an animal reservoir or emergence from an immunocompromised patient [109].

Based on 2 years of SARS-CoV-2 evolution, several observations can be made. First, gradual evolution, as seen in the second phase of the pandemic, would be expected to generate new antigenic drift variants, much like for influenza. Although this mechanism did not lead to significant antigenic drift in the short period of circulation of the Alpha to Gamma variants, it is reasonable to speculate that because the reported evolutionary rate is more rapid than that seen for influenza, it would lead to more frequent formation of new antigenic clades and require vaccine updates. A second potential mechanism, in line with the complex evolutionary history of Omicron, involves the periodic emergence of highly divergent strains from multiple possible reservoirs [110]. Because we do not clearly understand the emergence of Omicron, we cannot determine the expected frequency of such events in the future. Third, it is becoming clear that recombination is a mechanism for the generation of new SARS-CoV-2 variants [111]. New VOCs may arise due to recombination between SARS-CoV-2 strains cocirculating in the human population, or due to recombination between human and animal strains. During the pandemic, several new SARS-CoV-2 reservoirs have emerged via a reverse-zoonotic transmission process, most notably in minks and deer [112,113]. Given the multiple possible evolutionary routes that SARS-CoV-2 can take, we must maintain global epidemiological and genomic surveillance in a variety of hosts (i.e., a One Health approach) and have systems in place to rapidly update SARS-CoV-2 vaccines.

### Future studies

Several research areas should be strengthened to anticipate the endemicity patterns of SARS-CoV-2 on a population level, including genomic surveillance, longitudinal cohort-based approaches that combine epidemiologic and immunologic measurements, predictive computational models of SARS-CoV-2 evolution, and studies of recombination and viral mixing at the human–animal interface. We describe the potential value of these studies below.

#### Genomic surveillance

Using genomic surveillance of circulating viruses, researchers can track the emergence of new mutations and recombinant strains, identify new variants, monitor the relative prevalence of different variants within global and local populations, and evaluate genotype-to-phenotype changes (including severity, vaccine effectiveness, immune escape, and transmissibility). In the years ahead, it will be critical to analyze the circulation of virus strains in human, domestic and peri-domestic animals (e.g., deer) [112] to understand the role of nonhuman animal reservoirs in the emergence of new SARS-CoV-2 strains. Combined with collaborative genomic surveillance efforts between viral ecologists and veterinarians to study viral mixing at the human–animal interface, in vitro studies and animal experiments exploring the biology of SARS-CoV-2 recombination will be essential to understand how recombination plays into the path and pace of SARS-CoV-2 evolution. Further, global genomic surveillance needs to continue with a strengthened focus on LMICs, including in remote regions; it is worth noting that genomic surveillance teams in Botswana and South Africa first discovered the Omicron variant [114,115]. Genomic surveillance data directly impact vaccine updates and the need to implement new public health interventions [116].

An important aspect of monitoring new variants should include real-time assessment of clinical severity and vaccine effectiveness; here, it is important to link variant genetic characterization with patient vaccine history, medical background, and clinical outcomes. For example, a globally representative set of sentinel hospitals could be leveraged to monitor changes in the proportion of hospitalized patients requiring intensive care and in the hospital death rate as new variants arise. In addition to variation in virus sequences, there may be genetic predisposition for severe disease among particular hosts. Genome-wide association studies, which look for host genome differences between, for example, critically ill and mildly ill COVID-19 patients, can identify genetic risk factors for severe disease and help us learn more about the immunologic mediators of SARS-CoV-2 virulence [117,118].

#### Household studies and contact tracing

Data obtained from contact tracing and household transmission studies have been extremely useful for studying chains of transmission, kinetics of viral shedding, behaviors associated with increased risk of infection, and the effect of the vaccine on infection and transmission [119–122]. For example, the PHIRST household study found that in South Africa, attack rates were highest in participants aged 13 to 18 and that persons with HIV who were not virally suppressed were more likely to have symptomatic COVID-19 and to shed SARS-CoV-2 for a longer period of time compared to HIV-negative individuals [120]. Household studies will remain useful throughout the endemic period to monitor changes in transmission in well-described settings.

#### Prospective cohort studies

Longitudinal cohort studies have provided invaluable data on various aspects of the COVID-19 pandemic, including the age-dependent nature of SARS-CoV-2 infection risk; the persistence of SARS-CoV-2 antibodies; the loss of clinical protection and rate of reinfection over time; the risk of infection to healthcare workers; and the rates of Long COVID [72,119,120,123,124]. Cohorts can range dramatically in size, scope, and duration depending on whether they are prospective or retrospective (e.g., the ongoing prospective HIVE cohorts obtain infection and serologic data from approximately 1,000 individuals per year [60,82], while a retrospective cohort study recently analyzed long COVID data from the electronic health records of over 200,000 patients [124]). An understanding of the long-term path of SARS-CoV-2 will require investment in decades-long prospective cohort studies that regularly collect respiratory specimens and serum samples and where participants may accumulate multiple exposures to SARS-CoV-2 variants and vaccination episodes, and experience breakthrough infections due to changing levels of immunity.

Several cohort studies conducted during the pandemic are worth noting. Multiyear household respiratory virus cohorts, such as the HIVE study in Michigan [60] and the PHIRST study in South Africa [120], and the influenza-dengue cohorts in Nicaragua [36], are good models to analyze attack rates, viral shedding, chains of transmission, immunity and reinfection over time, and with different variants. For example, the data obtained from longitudinal infection testing and repeat antibody measurements are critical in determining the long-term risk of reinfection and the timeline on which the SARS-CoV-2 vaccines will need to be updated. Further, repeated serological measurements taken from a longitudinal cohort can be used to generate antibody landscapes, which can help elucidate the relevance of original antigenic sin (and possibly back-boosting) to the long-term path of SARS-CoV-2 [93]. These well-defined multiyear cohorts can also be leveraged for cellular immunology research and provide a better understanding of the mechanisms of B- and T-cell adaptation to viral evolution over time. It is important for cohorts to include participants from multiple age groups to compare patterns of immunity and infection between children and adults. Finally, cohorts can also examine whether it is possible to have cross-reactivity and/or cross-protection between SARS-CoV-2 and other viral antibodies. In the HIVE study, for example, researchers found little cross-reactivity between HCoV antibodies and SARS-CoV-2 spike proteins [82]. These cohorts can be leveraged to compare the cross-reactivity, clinical presentation, and potentially interdependent transmission dynamics of endemic SARS-CoV-2 and the seasonal coronaviruses. Rapid integration of SARS-CoV-2 research into existing respiratory virus focused cohorts has shown the power of these research platforms for collecting multi-pathogen information without having to identify a new population or having to design new study protocols in the midst of a pandemic [29,60].

Birth cohorts, which follow a group of people born around the same time, can help answer questions about how SARS-CoV-2 immunity builds up over time in a naive individual and how it compares to earlier birth cohorts, i.e., exploring the concept of “original antigenic sin” for SARS-CoV-2 [87]. Birth cohorts are essential to continue research on the immune response in immunologically naive individuals because at present a majority of the global population has been exposed to SARS-CoV-2 through either natural infection or vaccination.

#### Infection and vaccination cohorts

Useful information can also be gleaned from smaller and more targeted cohorts. Infection and vaccination cohorts typically follow 20 to 50 individuals and bleed participants pre- and post-antigen exposure. These cohorts can generate a large amount of important serological data, such as rates of seroconversion and levels of cross-reactive antibodies, but they are not necessarily representative of population-level immunological patterns [87]. Finally, longitudinal cohorts focused on individuals with prolonged SARS-CoV-2 shedding, particularly among immunosuppressed individuals, could shed light on the contribution of this population subgroup to viral evolution [125].

#### Computational modeling

Scientific advances in computational modeling are also needed to anticipate the evolution of SARS-CoV-2 and implement timely vaccine updates, guided by recent developments in predictive models of influenza antigenic drift and strain fitness [126]. Predictive approaches must be cautious in light of Omicron, as we do not yet know whether this variant is an evolutionary outlier or represents a parallel mechanism of SARS-CoV-2 evolution [127]. New developments in antigenic cartography, which relies on analyzing the cross-neutralizing antibody responses against viral variants to track antigenic changes, will be important to document, and perhaps one day predict, evolutionary changes [107].

## Conclusions

We conclude this review with several key takeaways. First, the periodicity of SARS-CoV-2 outbreaks during the endemic period will be driven by the contribution of reinfections to transmission dynamics, the rate of influx of new susceptibles (due to waning immunity, lack of vaccination, and new births), and the response to environmental forcing and seasonal contacts (schools and holidays). Second, the age distribution of SARS-CoV-2 is unlike many other well-studied viruses, such as influenza, measles, and dengue, due to biological factors that reduce susceptibility to infection and attenuate disease severity in children. Third, while the 4 seasonal coronaviruses are thought to predominantly infect young children and generally cause mild respiratory disease, cohort and surveillance studies have shown that the incidence of seasonal coronavirus infection does necessarily decline with age. Fourth, the durability of SARS-CoV-2 immune protection against infection and transmission, after repeat exposures to vaccination and antigenically distinct variants, will shape the epidemiology of future outbreaks. Subtle immunological interactions between responses to different coronaviruses could also modulate SARS-CoV-2 endemicity patterns. Finally, potential mechanisms of SARS-CoV-2 evolution include gradual evolution leading to antigenic drift, the periodic emergence of highly divergent strains (e.g., Omicron) from multiple possible reservoirs, and recombination generating new variants. How much each mechanism will contribute to the generation of new variants in the endemic phase cannot be predicted, nor can the periodicity of variant emergence. In light of these uncertainties, we see many opportunities in the coming years for experimental, observational, and computational research to begin to address these questions and provide insights that may be transferable to other acute respiratory infections. We hope that this review will inspire researchers to study the invasion and epidemiology of the seasonal coronaviruses using archeo-epidemiologic, serologic, and phylodynamic methods, as well as to expand prospective cohort studies, serologic testing, and large-scale genomic surveillance to address questions about duration of immunity, viral shedding, reinfection, and variant dynamics. We believe such studies will elucidate aspects of the inevitable shift from a pandemic to endemic SARS-CoV-2 that can guide future public health and policy efforts.
